# First-Principles Investigation of Structural, Mechanical, Electronic and Optical Properties of Ba_2_MReO_6_ (M = Li, Na, K, and Rb) Double Perovskites

**DOI:** 10.3390/ijms27146186

**Published:** 2026-07-10

**Authors:** Marcin Gackowski, Katarzyna Mądra-Gackowska, Muhammad Usman Khan, Łukasz Szeleszczuk

**Affiliations:** 1 Department of Toxicology and Bromatology, L. Rydygier Collegium Medicum in Bydgoszcz, Nicolaus Copernicus University in Torun, 2 Jurasza Street, 85-089 Bydgoszcz, Poland; marcin.gackowski@cm.umk.pl; 2 Department of Geriatrics, L. Rydygier Collegium Medicum in Bydgoszcz, Nicolaus Copernicus University in Torun, 9 Skłodowskiej Curie Street, 85-094 Bydgoszcz, Poland; katarzyna.madra@cm.umk.pl; 3 Department of Chemistry, University of Okara, Okara 56300, Pakistan; usmankhan@uo.edu.pk; 4 Department of Organic and Physical Chemistry, Medical University of Warsaw, 1 Banacha Street, 02-097 Warsaw, Poland

**Keywords:** Ba_2_MReO_6_, double perovskites, HSE06, band gap, optical properties, solar-cell applications, mechanical stability

## Abstract

The growing demand for efficient, stable, and environmentally friendly materials for next-generation optoelectronic and photovoltaic applications has attracted significant interest in double perovskite compounds. First-principles density functional theory (DFT) calculations were performed to systematically investigate the structural, mechanical, electronic, and optical properties of Ba_2_MReO_6_ (M = Li, Na, K, and Rb) double perovskites. Structural optimization confirms that all compounds crystallize in the cubic *Fm3̅m* symmetry. The thermodynamic and geometric stability of the series is checked with negative formation energies and tolerance factor analyses (*t*, *μ*, *τ*). Mechanical analysis confirms that all compounds are mechanically stable; Ba_2_LiReO_6_ is the stiffest, while Ba_2_RbReO_6_ shows moderate stiffness with the highest ductility. Furthermore, ab initio molecular dynamics (AIMD) simulations at room temperature confirm the dynamical stability of all compounds, with negligible fluctuations in total energy under thermal conditions. The calculated band structures using both GGA-PBE and HSE06 hybrid functionals reveal that all compounds possess indirect band gaps, with HSE06 values of 2.236 eV for Ba_2_LiReO_6_, 2.133 eV for Ba_2_NaReO_6_, 2.116 eV for Ba_2_KReO_6_, and 1.395 eV for Ba_2_RbReO_6_. Optical measurements indicate that it is highly polarizable by dielectric polarizability, has high absorption coefficients (approximately 10^6^ cm^−1^), and has large optical conductivity in the UV, with large inter-band interactions between 2 and 4 eV. The suitable band gap and favorable optical characteristics suggest that Ba_2_RbReO_6_ is the most promising candidate for photovoltaic and solar-cell applications.

## 1. Introduction

In recent years, the development of eco-friendly, cost-effective, and earth-abundant materials suitable for large-scale production has attracted significant research interest within the photovoltaic community. Among current solar-cell technologies, first-generation silicon-based solar cells dominate the world market, with more than an 80% market share and power conversion efficiencies (PCEs) exceeding 23% [1]. However, their high production and installation costs have prompted researchers to develop third-generation photovoltaic technologies that use cheaper, more sustainable, and abundant materials. Here, lead halide perovskite-based solar cells have also become promising solar-cell alternatives with PCEs over 20% and comparatively simple fabrication processes [2,3,4]. Severe stability limitations offset their high efficiency—indeed, they can be easily destroyed by humidity, oxygen, and ultraviolet light and are therefore limited in practical use [5]. Moreover, lead toxicity is not only hazardous to the environment but also to human health. Attempts to substitute Pb with other metals, such as Sn, have not been very successful; for example, CH_3_NH_3_SnI_3_ has a PCE of approximately 6% but is unstable in air because the Sn^2+^ species is oxidized to Sn^4+^. As a result, a significant challenge is finding stable, lead-free substitutes for perovskites [6].

The general formula of perovskite materials is ABC_3_, and it has been studied in many applications, such as fuel cells, electrochemical sensors [7], scintillators, and X-ray sensors [8], and photovoltaic cells [9]. Simple perovskites are normally cubic with space group Fm-3m (225) [10], the A-site cation in a 12-fold cuboctahedral coordination with anions surrounding it, and the B-site cation in an octahedral coordination. In addition to simple perovskites, double perovskites (A_2_BB′C_6_) have also been of particular interest due to their higher tuneability and stability [11]. These structures contain A, B, B′, and C atoms that occupy the 8c, 4a, 4b, and 24e Wyckoff positions, respectively [12].

In recent years, the optoelectronic properties of double perovskites have attracted considerable attention due to their chemically flexible crystal framework, which allows the incorporation of a wide variety of metal cations [13,14,15]. Organic and Pb-based double perovskites, such as Pb_2_ScBiO_6_ and Cs_2_PbGeF_6_, exhibit promising photovoltaic properties and low material costs; however, the toxicity of Pb remains a major challenge for their practical applications [16,17]. To break this, Slavney et al. have prepared the inorganic compound Cs_2_AgBiBr_6_ and highlighted its potential applications in optoelectronics and photovoltaics [12,17]. Although Cs_2_AgBiBr_6_ has an appropriate bandgap, its low absorption efficiency stems from its indirect nature. Likewise, Volonakis et al. investigated Cs2InAgCl6 and found its direct bandgap to be 3.3 eV, which could be tuned via halide mixing [18]. Moreover, as Kangsabanik et al. and Chakraborty et al. both stated, compared to simple perovskites, double perovskites are more structurally stable and scalable, and thus have a high chance of becoming next-generation solar cells [19,20]. Geisz et al. achieved a record solar cell efficiency of 47.1%, 11.1% higher than the previous record for thin-film solar cells [21]. Furthermore, recent first-principles studies have extensively explored the multifunctional properties of double perovskites, including half-metallic ferromagnetism in C- and N-doped Sr_2_MSbO_6_ (M = Al, Ga), optoelectronic and thermoelectric performance of lead-free Rb_2_InSbX_6_ (X = Cl, Br), and the suitability of Rb_2_LiBiX_6_ (X = Cl, F, Br, I) compounds for optoelectronic and solar cell applications, highlighting the growing interest in environmentally friendly and high-performance double perovskite materials [22,23,24].

Although these improvements have been made, the primary issues with the application of double perovskites in photovoltaics are the large, usually indirect, band gaps that prevent strong optical absorption. Meng et al. demonstrated that due to the inversion symmetry in most halide double perovskites, an optical forbidden transition occurs [25]. Nevertheless, recent computational studies have identified several double perovskite compositions, such as Cs_2_(In, Tl)^+1^(Sb, Bi)^+3^X_6_, that exhibit direct band gaps and promising theoretical power conversion efficiencies, highlighting viable pathways to overcome these intrinsic limitations [26].

In this work, we systematically investigate the structural, electronic, optical, mechanical, and dynamical properties of Ba_2_MReO_6_ (M = Li, Na, K, Rb) double perovskites using first-principles calculations. Our results demonstrate that these compounds are not only structurally and thermodynamically stable, but also dynamically stable, as confirmed by ab initio molecular dynamics simulations at room temperature. Although some studies on double perovskite materials have been reported in the literature, comprehensive theoretical investigations of the Ba_2_MReO_6_ (M = Li, Na, K, Rb) family remain limited. Therefore, the present work provides a useful foundation for understanding their intrinsic properties and offers valuable guidance for future experimental efforts to develop stable and efficient perovskite-based solar materials.

## 2. Results and Discussion

### 2.1. Structural Properties

**Figure 1:**
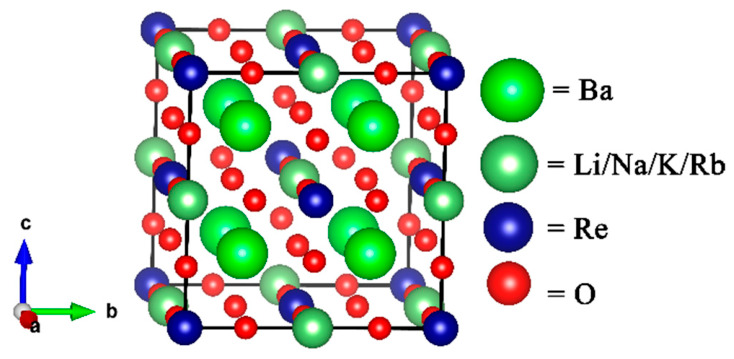
Crystal structure of Ba_2_MReO_6_ (M = Li, Na, K, and Rb) double perovskites.

Double perovskites are a subclass of perovskites in which either the A- or B-site of the conventional ABO_3_ lattice is occupied by two different types of cations (Figure 1). This gives rise to the general formulas A′A″B_2_O_6_ (double A-site) or A_2_B′B″O_6_ (double B-site). Since the A-site cations primarily act as electron donors to the $\left[BO_{6}\right]$ framework, while the B-site cations strongly influence the physical properties of perovskites, the term *double perovskite* generally refers to double B-site perovskites (A_2_B′B″O_6_). The crystal structure of a double perovskite A_2_B′B″O_6_ is determined by the ordering of the B′ and B″ cations within the B-sublattice. Differences in charge and/or ionic size between B′ and B″ ions favor an ordered arrangement, as this reduces both Madelung energy and lattice strain [27,28].

Oxide double perovskites Ba_2_MReO_6_ (M = Li, Na, K, Rb) that will be investigated in the present paper are face-centered cubic (fcc) crystallized and with the space group Fm3̅m (No. 225). The [MO_6_] and [ReO_6_] octahedra share corners in this structure to form a three-dimensional network, and the interstitial voids are occupied by the Ba^2+^ cations, which provide structural stability. Table 1 summarizes the optimized lattice parameters, the lattice constant (a0), the unit cell volume (V), and the formation energy (*E_f_*). Since experimental crystallographic data are available only for the parent compound Ba_2_LiReO_6_, the calculated structural parameters for the studied materials agree well with the reported values. All other parameters have been obtained from first-principles theoretical calculations.

Both thermodynamic stability and dynamical stability should be tested to determine the stability of these compounds. Because of the computational intensity of the calculation of AIMD, an initial check of thermodynamic feasibility is carried out with several descriptors: total energy, formation energy, Goldschmidt tolerance factor (*t*), octahedral factor (*μ*), and the recently introduced tolerance factor (*τ*). Formation energy (*E_f_*) is calculated with the following equation [31]:

$$\;E_{f}=\frac{E_{{Ba}_{2}{MReO}_{6}-n_{Ba}\times E_{Ba}-n_{M}\times E_{M}}-n_{Re}\times E_{Re}-n_{O}\times E_{O}}{N}$$

where $E_{{Ba}_{2}{MReO}_{6}}\;$ is the total energy of the unit cell of the double perovskite, *E_Ba_*, *E_M_*, *E_Re_*, and *E_O_* are the total energies of the individual atom species (Ba, Li/Na/K/Rb, Re, and O, respectively), and N atoms is the total number of atoms in the unit cell of the formula. The negative values of the *E_f_* values (Table 1) confirm that all the examined compounds are thermodynamically stable and can, in theory, be synthesized.

The stability of perovskites is traditionally evaluated using the Goldschmidt tolerance factor (*t*) and the octahedral factor (*μ*). Recently, Bartel et al. [32,33,34] proposed an improved tolerance factor (*τ*) that offers greater predictive accuracy. These factors are defined as:

$$\;t=\frac{\left(R_{A}+R_{X}\right)}{\sqrt{2}\left(R_{B}+R_{X}\right)}$$

$$\;µ=\frac{R_{B}}{R_{X}}$$

$$\;\tau =\frac{R_{X}}{R_{B}}-n_{A}\left(n_{A}-\frac{R_{A}/R_{B}}{In\left(R_{A}/R_{B}\right)}\right)$$

where $R_{A}$, $R_{B}$, and $R_{X}$ are the Shannon ionic radii of A-, B-, and X-site ions, respectively, and n_A_ is the valence of the A-site cation. In these double perovskites, R_B_ is the mean ionic radius of Re^5+^ and the alkali cation M^+^. Typical perovskite crystal structures are expected to meet 0.81 < t < 1.11, 0.60 < μ < 0.66, and τ < 4.18, with the latter the stability threshold of the elastic structure [35]. Table 2 presents a list of calculated tolerance factors of the studied compounds.

Table 2 results indicate that the tolerance factor (*t*) decreases gradually with the ionic radius of the alkali metal cation, Li^+^ to Rb^+^, and the octahedral factor (*μ*) increases, and the new tolerance factor (*τ*) increases. The whole *t*, *μ*, and *τ* range falls within the known stability domains, indicating that Ba_2_MReO_6_ (M = Li, Na, K, Rb) perovskites are structurally stable and likely to adopt the cubic Fm3̅m crystal structure.

### 2.2. Dynamical Stability

**Figure 2:**
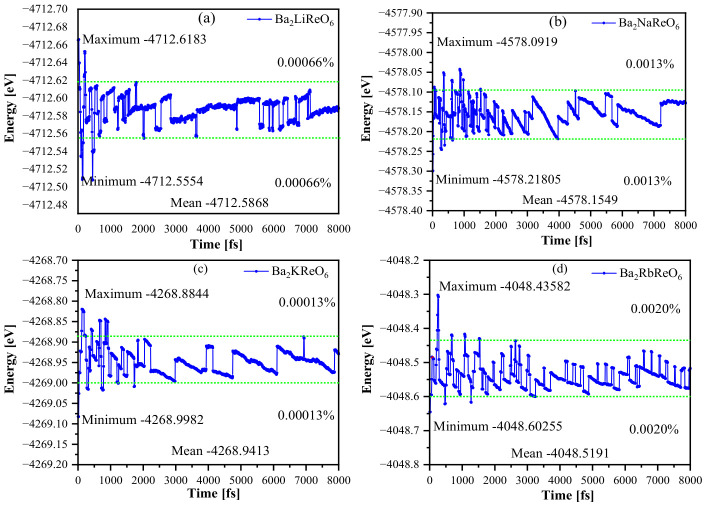
Ab Initio Molecular Dynamics (AIMD) simulations at room temperature (300 K) for total energy fluctuations with time for (**a**) Ba_2_LiReO_6_, (**b**) Ba_2_NaReO_6_, (**c**) Ba2KReO6, and (**d**) Ba_2_RbReO_6_ compounds.

Figure 2 illustrates the ab initio molecular dynamics (AIMD) simulations carried out at 300 K to assess the finite-temperature dynamical stability of the Ba_2_MReO_6_ (M = Li, Na, K, Rb) double perovskites. The evolution of total energy over 8 ps of simulation time shows that all compounds maintain stable energy oscillations around their respective mean values, with no signs of systematic energy drift, phase transitions, or structural degradation. The calculated energy fluctuations are extremely small, remaining within 0.00066% for Ba_2_LiReO_6_, 0.0013% for Ba_2_NaReO_6_ and Ba_2_KReO_6_, and 0.0020% for Ba_2_RbReO_6_, indicating excellent thermal robustness. These minor fluctuations arise from anharmonic lattice vibrations induced by thermal motion and are characteristic of dynamically stable crystalline solids at room temperature.

A slight increase in the magnitude of energy fluctuations is observed as the alkali-metal ion changes from Li to Rb, which can be attributed to the larger ionic radius and increased mass of the heavier alkali cations, thereby enhancing lattice flexibility and strengthening anharmonic effects. Nevertheless, the absence of any abrupt energy jumps or long-term instability confirms that the ReO_6_ octahedral framework remains intact throughout the simulation. The persistence of a well-defined mean energy further indicates that the structures are equilibrated and mechanically resilient under thermal excitation. Collectively, these AIMD results suggest that all Ba_2_MReO_6_ compounds remain dynamically stable at room temperature over the simulated timescale, supporting their potential suitability for experimental synthesis and device applications.

It should be noted that phonon dispersion calculations were not included in the present study; therefore, the assessment of structural and dynamical stability mainly relies on the calculated formation energy, tolerance factors, octahedral factor, and AIMD simulations. Although these results strongly support the stability of the Ba_2_MReO_6_ compounds, future investigations involving phonon spectrum analysis would provide a more comprehensive understanding of their lattice dynamical stability.

### 2.3. Electronic Properties

**Figure 3:**
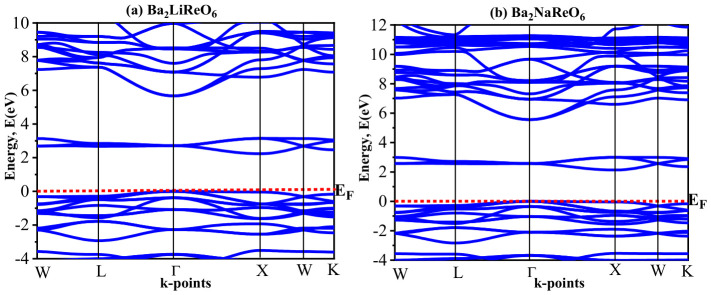

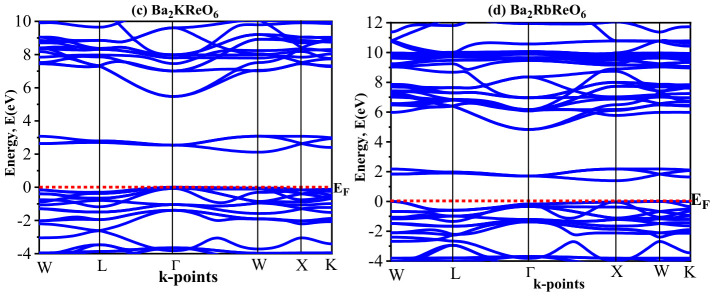
Band structure of Ba_2_MReO_6_ (M = Li, Na, K, and Rb) double perovskite.

The electronic properties of the Ba_2_MReO_6_ (M = Li, Na, K, and Rb) perovskites, their band gap energies, and the nature of their band structures are important in dictating whether they can be used in optoelectronic and photovoltaic applications. Table 3 summarizes the calculated band gaps for all compounds, and the band structures show a well-defined separation between the valence band (VB) and conduction band (CB). The GGA-PBE and HSE06 exchange-correlation functionals have been used in computing the electronic band gaps. In GGA-PBE, the calculated band gaps are 1.82 eV (experimental), 1.955 eV (calculated) of Ba_2_LiReO_6_, 1.843 of Ba_2_NaReO_6_, 1.786 eV of Ba_2_NaReO_6_, and 1.110 eV of Ba_2_RbReO_6_. Hybrid HSE06, as it would be expected, predicts slightly larger and more accurate gaps of 2.236 eV, 2.133 eV, 2.116 eV, and 1.395 eV for Ba_2_LiReO_6_, Ba_2_NaReO_6_, Ba_2_KReO_6_, and Ba_2_RbReO_6_, respectively. The band-structure analysis of all the compounds has confirmed an indirect band gap, as indicated by the dispersion of both the conduction and valence bands (Figure 3).

Examining the variation in the band gap with the ionic radius of the M-site cation reveals a clear trend: as the ionic radius increases along the series Li < Na < K < Rb, the band gap systematically decreases. Therefore, the largest band gap is in the case of Ba_2_LiReO_6_, and the smallest in the case of Ba_2_RbReO_6_. This flexibility in the electronic structure shows the potent role of cation size in the optical and electronic properties of halide double perovskites. The variable band gap (about 1–2 eV) suggests that Ba_2_MReO_6_ compounds can be tailored for a variety of optoelectronic applications. The band gaps of the materials should be in the order of 2 eV to enable visible-light harvesting and closer to 1 eV to extend absorption into the near-infrared range. Hence, the Ba_2_MReO_6_ family is a promising class of materials for the next generation of optoelectronic and energy-conversion devices.

**Figure 4:**
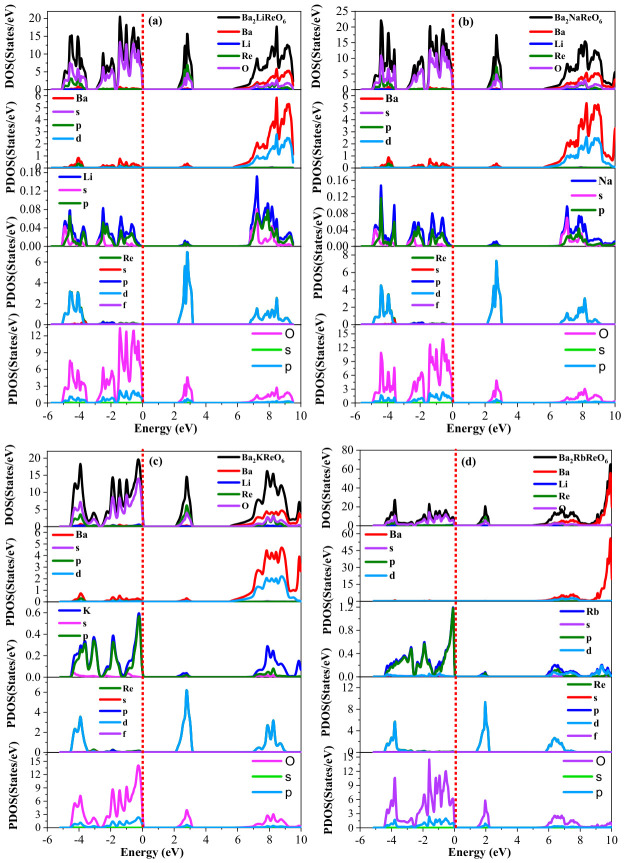
Total and partial density of states of Ba_2_MReO_6_ (M = Li, Na, K, and Rb) double perovskite. (**a**): Ba_2_LiReO_6_, (**b**): Ba_2_NaReO_6_, (**c**): Ba_2_KReO_6_, (**d**): Ba_2_RbReO_6_.

Figure 4a–d shows the total density of states (TDOS) of the Ba_2_MReO_6_ (M = Li, Na, K, Rb) double perovskites, which provide further information on the electronic structure beyond the band-structure results. The energy range of the DOS profiles is −6 to 10 eV, with EF at 0 eV. The overall DOS of any compound is an unambiguous depiction of the distinct separation existing between the valence band (VB) and conduction band (CB) that proves the semiconducting nature of the compound, determined by the calculated band gaps. The VB region (between −6 eV and the *E_F_*) is mainly occupied by O-2*p* states that strongly hybridize with Re-5*d* orbitals. The strong *p–d* interaction is important in controlling the VB width and the overall electronic distribution. The deeper valence energies are primarily due to Ba-*s* and Ba-*p* states, and have a minor effect on the band edges.

The conduction band of all Ba_2_MReO_6_ compounds is mostly built upon Re-5*d* states, which are located in the lower section of the CB and play an important role in the optical transition behavior. Other contributions come from the O-2p orbitals, with higher conduction energy indicating strong covalency in the ReO_6_ octahedral structure. The alkali–metal cations (Li, Na, K, Rb) are characterized by low levels of electronic activity at *E_F_*. The Li-2*s*/2*p* and Na-3*s*/3*p* states of Ba_2_LiReO_6_ and Ba_2_NaReO_6_ are significantly dispersed along the edges of the VB and CB, suggesting that they do not interact with the ReO_6_ network. Likewise, deeper K-4*s* and 4*p* states are observed in Ba_2_KReO_6,_ and the Rb-5*s*/5*p* states in Ba_2_RbReO_6_ are far from the Fermi level, indicating that they are inert, ionic electrons.

Thus, the DOS and PDOS studies indicate that the electronic properties of the Ba_2_MReO_6_ series are determined primarily by the hybridization of O-2p and Re-5d orbitals. In contrast, the alkali-metal ions play a negligible role in the states near the Fermi level. This orbital distribution is consistent with the indirect semiconducting band character observed in the band-structure calculations. It underscores the central role of the ReO_6_ octahedra in shaping the electronic and optical characteristics of these double perovskites.

**Figure 5:**
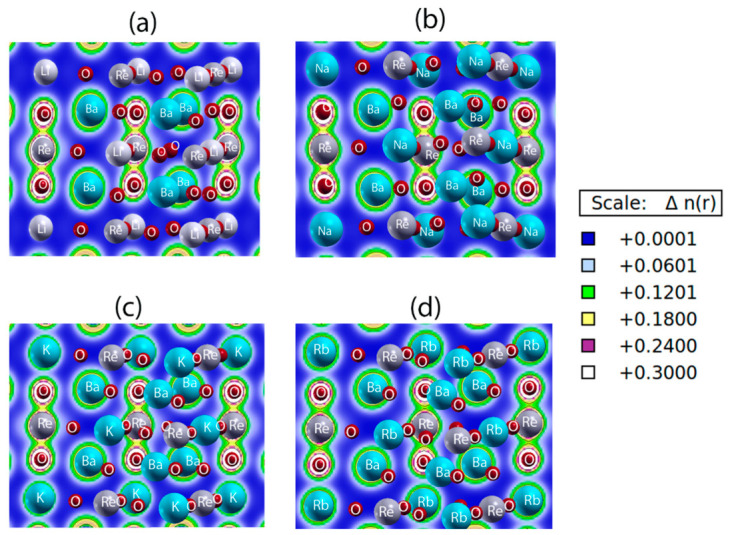
Charge density mapping of Ba_2_MReO_6_ (M = Li, Na, K, Rb) double perovskite. (**a**): Ba_2_LiReO_6_, (**b**): Ba_2_NaReO_6_, (**c**): Ba_2_KReO_6_, (**d**): Ba_2_RbReO_6_.

In Figure 5a–d, electron charge density difference maps of Ba_2_MReO_6_ (M = Li, Na, K, Rb) would reveal differences in the distribution of electrons around the cations and oxygen atoms. In Ba_2_LiReO_6_ (Figure 5a), charge density is highly concentrated along Re-O bonds, and Li does not interact with oxygen, meaning that it is mainly ionically bonded with oxygen. In Ba_2_NaReO_6_ (Figure 5b), the density of the electrons around Na is more pronounced, indicating a stronger contact between M and O and a partial covalent bond. However, the number of Re-O bonds is also important. In the case of Ba_2_KReO_6_ (Figure 5c), K exhibits a considerable charge accumulation, which is indicative of increased hybridization and a partial covalent nature of K-O bonds, but still preserves the Re-O structure. Lastly, in Ba_2_RbReO_6_ (Figure 5d), Rb is the most densely populated alkali cation in the crystal, which implies that it shares many electrons and that it has predominantly mixed ionic and covalent bonds. In general, the A-site cation effect on M-O interactions is more covalent and stronger, and the ReO_6_ octahedra always maintain high electron density, which highlights their structural and electronic stability. It should be noted that spin–orbit coupling (SOC) effects were not considered in the present study. However, they may influence the electronic structure due to the presence of heavy Re atoms, warranting exploration in future investigations.

### 2.4. Mechanical Properties

The elastic constants of Ba_2_MReO_6_ (M = Li, Na, K, and Rb) were verified to determine the mechanical stability and the general mechanical strength. These constants are used to measure a material’s resistance to external mechanical deformation. The calculated values of *C*_11_, *C*_12_, and *C*_44_ are presented in Table 4. Based on Born stability criteria of a cubic system [36,37], a crystal at rest should have met the following requirements to be considered mechanically stable.

$$\;\left(C_{11}-C_{12}\right)>0,\;\left(C_{11}+{2C}_{12}\right)>0,\;C_{11}>0,\;C_{44}>0,\;C_{12}<B<C_{11}$$

These requirements are satisfied in all Ba_2_MReO_6_ compounds (Table 4), thereby validating their mechanical stability.

The bulk modulus (*B*), shear modulus (*G*), Young’s modulus (*Y*), and Poisson’s ratio (*ν*) were calculated using the Voigt–Reuss–Hill approximations [38,39]:

$$B=\frac{C_{11}+{2C}_{12}}{3},$$

$$G=\frac{\left(G_{V}+G_{R}\right)}{2},$$

$$\;Y=\frac{{9BG}_{V}}{3B+G_{V}}$$

$$\;v=\frac{3B-2G}{2(3B+2G)}$$

Here, *B* measures volume compression resistance, *G* measures shear deformation resistance, and *Y* measures overall stiffness [40]. Table 4 presents the summary that Ba_2_LiReO_6_ is the most stiff (Y = 216.35 Gpa) and Ba_2_RbReO_6_ is the least stiff (Y = 69.91 Gpa). A gradual reduction in *B*, *G*, and Y at Li → Na → K → Rb indicates a gradual weakening of the interatomic bonding, which is in agreement with the gradient of the ionic radius of the M-site cation.

**Figure 6:**
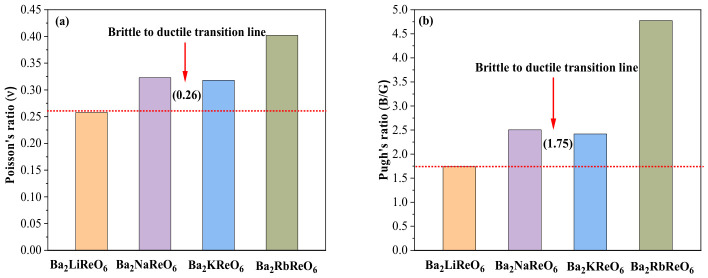
The mechanical aspects, including (**a**) Poisson’s ratio, (**b**) Pugh’s ratio of Ba_2_MReO_6_ (M = Li, Na, K, and Rb) compounds.

Poisson’s ratio and Pugh’s ratio, which are the popular measures of plastic deformability, were used to assess the ductile or brittle character of the Ba_2_MReO_6_ compounds [41,42]. Ba_2_LiReO_6_ has low values of *ν* (0.258) and *B/G* (1.738), both below critical ductility thresholds, and thus it exhibits a brittle nature and is more prone to breaking under pressure. On the other hand, the values of and *B/G* in Ba_2_NaReO_6_, Ba_2_KReO_6_, and Ba_2_RbReO_6_ exceed the ductile limits, indicating that these materials can undergo plastic deformation and resist crack propagation. Such a change in brittle (Li) to increased ductility (Na, K, Rb) is in line with the lowering of the lattice as the ionic radius of M increases. The above mechanical trends are consistent with the behavior shown in Figure 6a,b, and it is clear that only Ba_2_LiReO_6_ is brittle, whereas the other compounds are ductile.

The anisotropic factor (*A*) is also important in the realization of the mechanical response and prediction of microscopic hardness. In the case of cubic systems, the evaluation of elastic anisotropy is through the use of Zener anisotropy index [43,44],

$$\;A=\frac{2C_{44}}{C_{11}+C_{12}}$$

For cubic crystals, A = 1 yields perfect isotropy. The calculated values of 1.193, 1.900, 0.762, and 3.813 of the compounds of Ba_2_LiReO_6_ Ba _2_NaReO_6_, and Ba_2_KReO_6_ and Ba_2_RbReO_6_, respectively, indicate high levels of elastic anisotropy in all the compounds. Greater deviation of unity means greater anisotropy, which may facilitate microcrack formation at the microscopic level [45,46].

The melting temperatures estimated in Table 4 also exhibit a clear correlation with the materials’ elastic behavior. The melting temperature of the series rises as Ba_2_KreO_6_ possesses the highest melting temperature (2163.05 K), whereas Ba_2_RbReO_6_ has the lowest (1344.33 K), which agrees with the variation in their elastic strengths. Generally, materials with high elasticity are more strongly bonded and therefore more thermally resistant. For cubic crystals, the melting temperature is frequently calculated using the empirical relation [47].

$$\;T_{m}=\left(553+5.91C_{11}\right)K$$

providing evidence that increased values of C_11_ directly lead to increased melting temperature.

The Debye temperature (D) is a key parameter that indicates the nature of lattice vibrations, the strength of bonds, and the thermal behavior of a material. It corresponds to the highest frequency of phonons in a crystal. It can be theoretically determined with the help of the average sound velocity (*V_m_*) determined by the elastic constants, especially the bulk modulus (*B*) and shear modulus (*G*) [48]:

$$\theta_{D}\;=\frac{h}{k_{B}}[(\frac{3n}{4\pi })N_{A}\rho /M]V_{m}\;$$

where h is Planck’s constant, kB is the Boltzmann constant, n is the number of atoms in one unit of formula, NA is the Avogadro number, ρ is the density, and M is the molar mass.

h is Planck’s constant, k_B_ is Boltzmann’s constant, *n* is the number of atoms per formula unit, *N_A_* is Avogadro’s number, *ρ* is the density, and *M* is the molar mass. The average sound velocity *V_m_* for a polycrystalline material is given by:

Vm=[13(1vl3+2vt3)]−13

with the longitudinal (*v_l_*) and transverse (*v_t_*) sound velocities determined from the elastic moduli and density as:

Vl=[(3B+4G)/3ρ]12and Vt=[G/ρ]12

In the case of Ba_2_MReO_6_ (M = Li, Na, K, and Rb), there is a systematic reduction in the *θ_D_* value with the size of the cation: Ba_2_LiReO_6_ (491.29 K) > Ba_2_KReO_6_ (436.43 K) > Ba_2_NaReO_6_ (393.65 K) > Ba_2_RbReO_6_ (259.16 K). This tendency is consistent with the calculated sound velocities, with Ba_2_LiReO_6_ having the largest longitudinal (6230.01 m/s), transverse (3554.77 m/s), and average (3950.49 m/s) velocities, indicating increased elastic interaction and a more rigid lattice. In contrast, Ba_2_RbReO_6_ exhibits slower velocities and D, indicating weaker bonding and a softer lattice. The observed difference in Debye temperature indicates that materials with smaller A-site cations are more likely to exhibit higher thermal conductivity and mechanical stability at lower temperatures, such as Ba_2_LiReO_6_ and Ba_2_KReO_6_ (Table 5).

### 2.5. Optical Properties

The optical behavior of a material is intrinsically linked to its dielectric function, which provides insights into its ability to interact with and harvest energy from visible light. Key optical characteristics such as the absorption coefficient *α(ω)*, refractive index *n(ω)*, and reflectivity *R(ω)* are derived from the dielectric function *ε(ω)* [49]. In the case of cubic systems, the dielectric has the property of isotropy, which means that the dielectric tensor is reduced to *ε_xx_ = ε_yy_ = ε_zz_ = ε*. Dielectric function may be broken down into real and imaginary components, *ε*_1_*(ω),* which is a measure of polarizability, and *ε*_2_*(ω)*, the measure of light absorption [50]. Peaks in *ε*_1_*(ω)* correspond to resonance frequencies where maximum light scattering occurs, while *ε*_2_*(ω)* reflects interband transitions and the material’s absorption behavior [51,52]. It is important to note that SLME calculations were not performed in this study; however, incorporating SLME analysis in future work would provide a more detailed and quantitative assessment of the photovoltaic efficiency of the Ba_2_MReO_6_ compounds.

**Figure 7:**
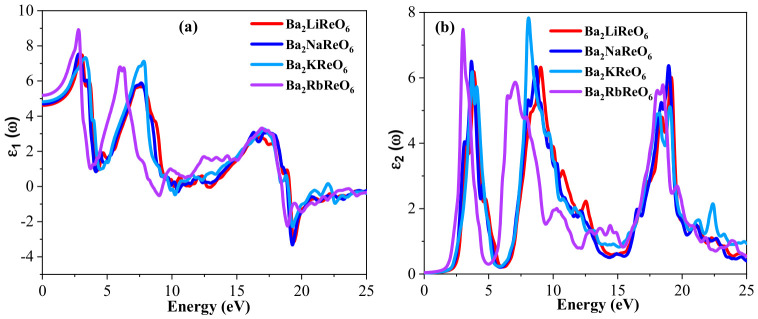

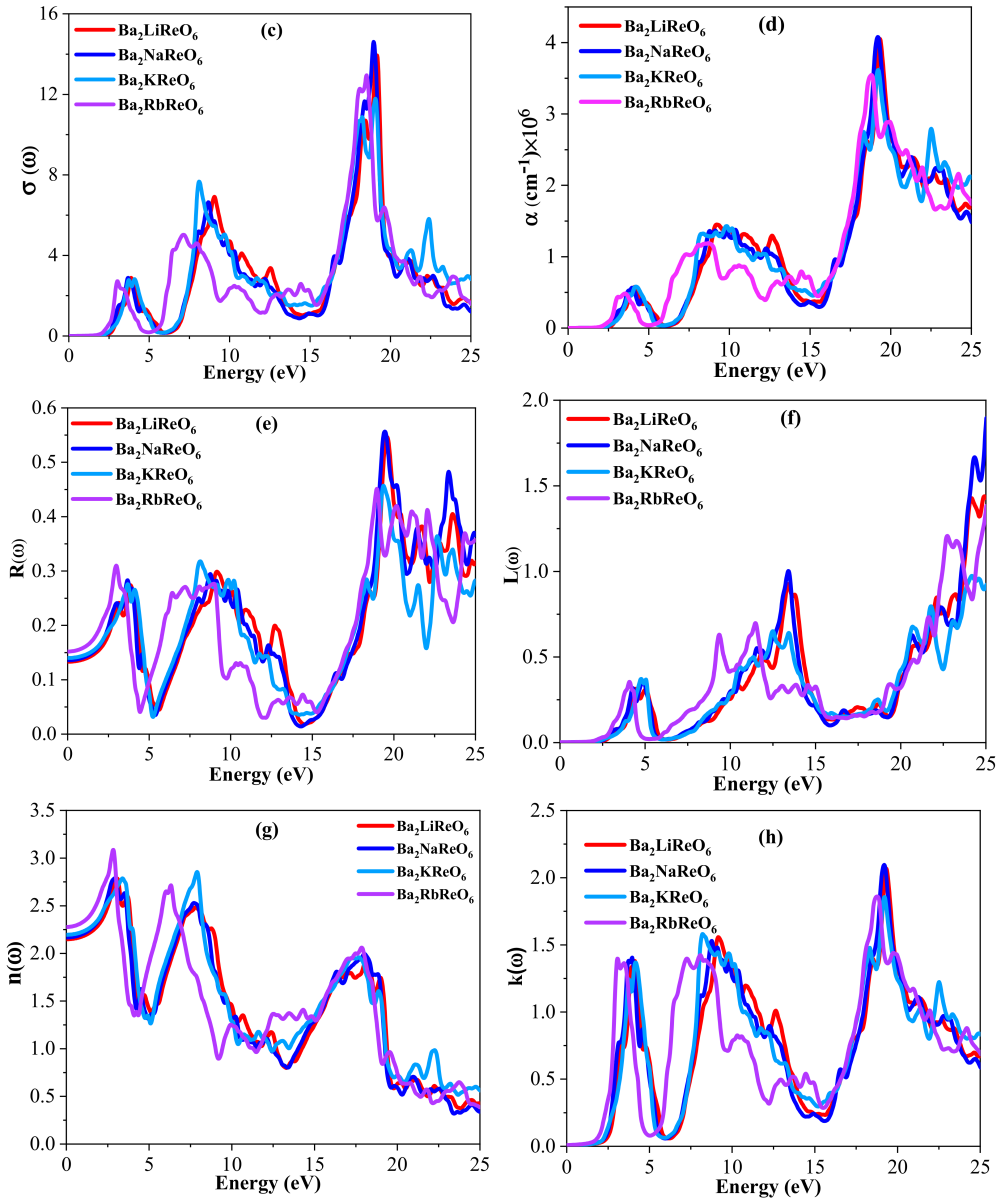
Calculated optical parameters of Ba_2_MReO_6_ (M = Li, Na, K, and Rb) double perovskite. (**a**): ε_1_(ω), (**b**): ε_2_(ω), (**c**): σ(ω), (**d**): ε_α_(ω), (**e**): R(ω), (**f**): L(ω), (**g**): n(ω), (**h**): k(ω).

The actual part of the dielectric function, which is labeled as ε_1_(ω), is shown in Figure 7a. The true fraction ε_1_(ω) of the dielectric constant of Ba_2_LiReO_6_, Ba_2_NaReO_6_, Ba_2_KReO_6_, and Ba_2_RbReO_6_ has the static points of 4.57, 4.64, 4.86, and 5.19, respectively. The peaks of *ε*_1_*(ω)* are 7.48 at 3.03 eV, 7.61 at 2.76 eV, 7.34 at 3.36 eV, and 8.98 at 2.79 eV, respectively. Other peaks are also noticeable at higher energies, as shown in Figure 7a. The Penn model, $\varepsilon_{1}\left(0\right)\approx (\frac{\hbar \omega \rho }{E_{g}})$ [53], is used in order to determine the values of *ε*_1_*(*0*)* and *E_g_* of the material under study by determining the condition of dielectric function *ε*_1_*(*0*) >* 1.

The *ε*_2_*(ω)* defines the light absorption and the depth of penetration and is directly connected with the band-gap energy of the substance. It explains inter-band changes in electrons: as photon energy increases, electrons shift from the valence band, which is predominantly composed of O-2p orbitals, to the conduction band, which is composed of Re-5*d*/M-*s*,*p* orbitals. It is a transition observed as the initial peak in all compounds. The second peak is also associated with these transitions, as shown in Figure 6b. Moreover, the peaks in the energy spectrum in the range of 2.0–4 eV demonstrate the significance of using the studied compounds in solar cells and optoelectronic devices.

The optical conductivity *σ(ω)* is caused by the free carriers that are created as incident photons are absorbed in promoting the electrons in the valence band to the conduction band. The incoming radiation causes attenuation of part of the wave as it interacts with the material, resulting in similar spectral features in both absorbance and conductivity curves. Therefore, the maximum of *σ(ω)* is located in the same energy ranges as those of *ε*_2_*(ω)* as well as the absorption coefficient, *α(ω)*, which is presented in Figure 7c. In the case of Ba_2_MReO_6_ (M = Li, Na, K, and Rb), *σ(ω)* displays its first noticeable rise in the low-energy region around 3–5 eV, corresponding to the onset of inter-band transitions. More pronounced peaks occur within the 8–12 eV range, followed by the strongest conductivity response between 15 and 20 eV for all compounds. Among them, Ba_2_KReO_6_ and Ba_2_NaReO_6_ have the largest peak intensities, while Ba_2_R ReO_6_ has a relatively low conductivity, consistent with its lower bonding strength and lower transition probability. These high and broad conductivity characteristics indicate that Ba_2_MReO_6_ compounds exhibit a high degree of photon-induced charge-carrier generation, suggesting they can be used in high-energy optoelectronic and photonic applications [54].

The absorption coefficient *α(ω)* of the Ba_2_MReO_6_ (M = Li, Na, K, Rb) gives an insight into their capacity to absorb incident photons as illustrated in Figure 7d. The value of the coefficient *α(ω)* is also indicative of the extent to which light can be penetrated before it is completely attenuated, following the relation α = 4πk/λ [55]. Every compound has an absorption onset based on the fundamental electronic transitions of the compound, with the strength of absorption increasing as photons elevate electrons to the conduction band. There are two prominent absorption features in the 5–10 eV region, after which it sharply increases, peaking around 15–20 eV. The highest *α(ω)* is 4.6 × 10^6^ cm^−1^ at 19.22 eV in case of Ba_2_LiReO_6_, 4.7 × 10^6^ cm^−1^ at 19.19 eV in the case of Ba_2_NaReO_6_, 3.6 × 10^6^ cm^−1^ at 19.14 eV in the case of Ba_2_KReO_6_, and 3.54 × 10^6^ cm^−1^ at 18.81 eV, respectively. Such large absorption coefficients imply that photon–electron interactions are strong and that incident radiation is rapidly attenuated at higher energies. This gradual reduction in the absorption intensity of the Li-to-Rb peak can be attributed to the increasing ionic radius, which is usually associated with a minor decrease in band-gap energy and a reduced electronic transition probability.

The reflectivity *R(ω)* given in Figure 7e is a ratio of the incident radiation reflected by the surface of the Ba_2_MReO_6_ compounds. The reflectivity values of Ba_2_LiReO_6_, Ba_2_NaReO_6_, Ba_2_KReO_6_, and Ba_2_RbReO_6_ start at 0.130, 0.134, 0.141, and 0.152, respectively, at 0 eV, indicating low reflectivity in the low-energy region. The reflectivity slowly increases with photon energy and exhibits several oscillations between 5 and 12 eV, which are attributed to interband electronic transitions. There is a decrease in reflectivity in the 12–15 eV range, indicating increased photon absorption in the same range. Above 15 eV, all the compounds reflect sharply with the maximum in the high-energy region (18–22 eV). The peak reflectivity values are 0.54 at 19.66 eV for Ba_2_LiReO_6_, 0.55 at 19.45 eV for Ba_2_NaReO_6_, 0.46 at 19.36 eV for Ba_2_KReO_6_, and 0.45 at 18.94 eV for Ba_2_RbReO_6_. Among the series, Ba_2_RbReO_6_ shows slightly stronger fluctuations, whereas Ba_2_LiReO_6_ and Ba_2_NaReO_6_ exhibit comparatively smoother spectral profiles. The low reflectivity at lower energy levels indicates that the materials are highly efficient at penetrating photons, making them a good choice for optoelectronic and solar energy applications.

The energy loss curve *L(ω)* in Figure 7f is the amount of energy lost by the fast-moving electrons when they interact with the Ba_2_MReO_6_ compounds. There are extremely low-loss materials below 4 eV, and above 5-15 eV, plasmon resonances and interband electronic transitions dominate multiple prominent peaks. The strength of the rise in *L(ω)* is strong above 15 eV, where the compounds exhibit the most intense energy-loss intensities. *L(ω)* peaks in the high-energy range (20–25 eV), which are 1.4 in the case of Ba_2_LiReO_6_, 1.8 in the case of Ba_2_NaReO_6_, 0.9 in the case of Ba_2_KReO_6_, and 1.3 in the case of Ba_2_RbReO_6_. The spectral fluctuations of Ba_2_RbReO_6_ are more intense than those of other compounds, which have smooth loss profiles. The strong plasmon excitation and strong electron–photon interaction in these materials are evident in the pronounced peaks at higher energies.

Lastly, the extinction coefficient *k(ω)* and the refractive index *n(ω)* versus photon energy are plotted in Figure 7g and Figure 7h, respectively. All the compounds of Ba_2_MReO_6_ in the low-energy region have relatively large refractive indices, which decrease steadily with photon energy. This decrease points to a depolarization of the optical response and a decreasing dielectric response as the materials shift into the ultraviolet region, which is higher in energy. Even though the general behavior of all compounds is the same, there is a significant variation in peak strength and other positions, because the M-site alkali-metal cation affects them. The highest peaks of *n(ω)* occur around 2–3.5 eV, and the highest response was observed in Ba_2_RbReO_6_, which indicates an increase in optical polarizability and an increase in its reaction to incident light. Figure 7h shows extinction coefficient spectra with several strong absorption peaks spanning 3-15 eV, corresponding to inter-band electronic transitions. Ba_2_KReO_6_ and Ba_2_RbReO_6_ have the highest value of *k(ω)* close to the major absorption peaks, and this shows that they have a better photon-absorption capacity than the other compositions. In comparison, Ba_2_LiReO_6_ consistently exhibits a lower k() value, indicating reduced optical absorption. Generally, the methodical differences both in *n(ω)* and *k(ω)* indicate that replacement with heavier cations of alkali-metal improves the dielectric response and increases optical transition probabilities in Ba_2_MReO_6_ double perovskites. It should be noted that carrier effective masses, charge transport properties, and exciton binding energies were not considered in the present study and may be investigated in future work following this study [56]. Overall, despite their indirect band-gap nature, the strong optical absorption and favorable dielectric response observed in these compounds suggest that phonon-assisted optical transitions may still enable efficient light harvesting, highlighting their potential for future photovoltaic and optoelectronic applications.

## 3. Materials and Methods

The calculations in this paper were performed using density functional theory (DFT) with the Projector-Augmented-Wave (PAW) method and ultrasoft pseudopotentials, implemented in the Quantum ESPRESSO (QE) software package, version 7.4.1 [57]. To examine the structural, mechanical, electronic, and thermal characteristics of the compounds, the Generalized Gradient Approximation (GGA) with the Perdew–Burke–Ernzerhof (PBE) exchange-correlation functional was used. After performing a broad convergence test on both materials, it was found that the best computational parameters were a plane-wave cutoff energy of 60 Ry and a 10 × 10 × 10 Monkhorst-Pack k-point mesh. The total energy was stabilized to a range of 10^−6^ Ry to guarantee electronic energy convergence. To better treat the exchange-correlation effects, particularly for electronic and optical properties, the Heyd–Scuseria–Ernzerhof (HSE06) hybrid functional was used [58]. Spin–orbit coupling (SOC) effects were not included in the present calculations. Accordingly, all electronic structure results reported in this work correspond to scalar-relativistic calculations. In contrast, a detailed investigation of SOC-induced modifications to the electronic and optical properties is reserved for future studies. The energy strain method, implemented in the thermos_pw package [59] has been used to compute elastic constants. Ab initio molecular dynamics (AIMD) simulations were performed within QE to assess the thermal stability and time-dependent behavior of the compounds at finite temperatures.

## 4. Conclusions

The physical properties of Ba_2_MReO_6_ (M = Li, Na, K, and Rb) double perovskites were systematically investigated using first-principles DFT calculations in the present study. Thermodynamic stability is verified by structural optimization and negative formation energies. They have been assessed based on the Goldschmidt tolerance factor (t), octahedral factor (μ), and modern tolerance factor (τ) to confirm that every composition is within the perovskite stability range, to guarantee geometric stability. Mechanical analysis indicates that all compounds meet the Born criteria, ensuring good mechanical stability. In addition, ab initio molecular dynamics (AIMD) simulations at room temperature suggest the dynamical stability of the compounds over the simulated timescale, as evidenced by only negligible total-energy fluctuations under thermal conditions. The ratios of Pugh’s and Poisson’s justify that Ba_2_LiReO_6_ has a brittle nature, and Ba_2_NaReO_6_, Ba_2_KReO_6_, and Ba_2_RbReO_6_ have a ductile nature. The indirect-band-gap semiconductors are obtained after calculating the electronic structure (PBE and HSE06), with the Ba_2_RbReO_6_ system exhibiting the most appropriate band gap for absorbing visible light. Both DOS and charge-density analyses reveal that the energy and momentum distributions of the O-2*p* states dominate the valence bands, while those of the Re-5d states dominate the conduction bands. Optical properties include strong dielectric behavior, high absorption coefficients (~106 cm−1), and high conductivity; Ba_2_RbReO_6_ has the highest absorption. In general, the M-site cation is an effective means of altering the structural, mechanical, electronic, and optical properties. Among them, Ba_2_RbReO_6_ emerges as the most promising candidate for solar-energy and optoelectronic applications. The present findings provide a reliable theoretical foundation for the future design and development of high-performance double-perovskite materials.

## Figures and Tables

**Table 1:** Lattice constant (*a*), unit cell volume (*V*), and formation energy ($E_{f}$) for Ba_2_MReO_6_ (M = Li, Na, K, Rb) compounds. Exp—experimental.

Compounds	*a* (Å)	*V* (Å^3^)	*E_f_*	Ref
Ba_2_LiReO_6_	8.24 ^exp^ 8.1960	558.49 ^exp^ 550.56	−2.742 ^exp^ −2.567	[29,30] This study
Ba_2_NaReO_6_	8.2864	568.98	−2.288	This study
Ba_2_KReO_6_	8.4756	608.85	−1.967	This study
Ba_2_RbReO_6_	8.6276	642.19	−1.647	This study

**Table 2:** Shannon ionic radii (*r*), Goldschmidt tolerance factor (*t*), octahedral factor (*μ*), and new tolerance factor (τ) for Ba_2_MReO_6_ (M = Li, Na, K, Rb) compounds.

Compound	Ionic Radius of Cations (Å)	Ionic Radius of Anion (Å)	Tolerance Factor (*t*)	Octahedral Factor (***u***)	New Tolerance Factor (***τ***)
Ba_2_LiReO_6_	r(Ba^2+^) = 1.61, avg(Li^+^, Re^5+^) = (0.76 + 0.58)/2 = 0.67	*r*(O^2−^) 1.40	1.03	0.48	2.40
Ba_2_NaReO_6_	r(Ba^2+^) = 1.61, avg(Na^+^, Re^5+^) = (1.02 + 0.58)/2 = 0.80	*r*(O^2−^) 1.40	0.97	0.57	2.55
Ba_2_KReO_6_	r(Ba^2+^) = 1.61, avg(K^+^, Re^5+^) = (1.38 + 0.58)/2 = 0.98	*r*(O^2−^) 1.40	0.89	0.70	2.80
Ba_2_RbReO_6_	r(Ba^2+^) = 1.61, avg(Rb^+^, Re^5+^) = (1.52 + 0.58)/2 = 1.05	r(O^2−^) 1.40	0.87	0.75	2.95

**Table 3:** Calculated band gap values of Ba_2_MReO_6_ (M = Li, Na, K, and Rb) compounds. Exp.—experimental.

Compounds	Ba_2_LiReO_6_	Ba_2_NaReO_6_	Ba_2_KReO_6_	Ba_2_RbReO_6_
GGA PBE	1.82 ^exp^, 1.955	1.843	1.786	1.110
HSE06	2.236	2.133	2.116	1.395
Nature	Indirect	Indirect	Indirect	Indirect

**Table 4:** Calculated elastic constants (*C*_11_, *C*_12_, *C*_44_), the bulk modulus (*B*), the shear modulus (*G*), Young’s modulus (*Y*), Poisson’s ratio (*ν*), the Pugh’s ratio (*B/G*), the Zener anisotropy factor (*A*), and the melting temperature *T_m_*(K) for Ba_2_MReO_6_ (M = Li, Na, K, Rb) compounds.

Parameters		Ba_2_LiReO_6_	Ba_2_NaReO_6_	Ba_2_KReO_6_	Ba_2_RbReO_6_
Born stability	***C***_**11**_ (GPa)	252.44	188.43	272.42	133.89
	***C***_**12**_ (GPa)	97.85	113.23	111.69	111.64
	***C***_**44**_ (GPa)	92.25	71.46	61.26	42.42
	***C***_**11**_ − ***C***_**12**_ (GPa)	154. 59	75.2	160.73	22.25
	***C***_**11**_ + **2*C***_**12**_ (GPa)	448.14	414.89	495.8	357.17
Bulk modulus, *B* (GPa)		149.38	138.30	165.27	119.06
Shear modulus, *G* (GPa)		85.94	55.22	68.30	24.93
Young modulus, *Y* (GPa)		216.35	146.22	180.09	69.91
Poisson’s ratio, *ν*		0.258	0.323	0.318	0.402
Pugh’s ratio, *B/G*		1.738	2.504	2.419	4.775
Zener anisotropy index, ***A***		1.193	1.900	0.762	3.813
Melting Temperature (*T_m_*)		2044.94	1666.67	2163.05	1344.33

**Table 5:** Calculated density (*ρ*), transverse, longitudinal, average sound velocity (*v_t_*, *v_m_*, *v_l_*), and Debye temperatures (*θ_D_*) of Double Perovskite Compounds Ba_2_MReO_6_ (M = Li, Na, K, Rb) compounds.

Compounds	Ba_2_LiReO_6_	Ba_2_NaReO_6_	Ba_2_KReO_6_	Ba_2_RbReO_6_
ρ (kg/m^3^)	6800.96	6768.13	6500.62	6642.67
V_t_ (m/s)	3554.77	2856.36	3241.4	1937.26
V_m_ (m/s)	3950.49	3200.29	3629.12	2193.66
V_l_ (m/s)	6230.01	5595.75	6279.53	4788.26
θ_D_ (K)	491.29	393.65	436.43	259.16

## Data Availability

The original contributions presented in this study are included in the article. Further inquiries can be directed to the corresponding author.
